# Omega-3 Fatty Acids Survey in Men under Active Surveillance for Prostate Cancer: from Intake to Prostate Tissue Level

**DOI:** 10.3390/nu11071616

**Published:** 2019-07-16

**Authors:** Hanane Moussa, Molière Nguile-Makao, Karine Robitaille, Marie-Hélène Guertin, Janie Allaire, Jean-François Pelletier, Xavier Moreel, Nikunj Gevariya, Caroline Diorio, Patrice Desmeules, Bernard Têtu, Benoît Lamarche, Pierre Julien, Vincent Fradet

**Affiliations:** 1 Oncology Axis, Centre de recherche du CHU de Québec – Université Laval, QC G1R 3S1, Canada; 2 Institute of Nutrition and Functional Foods (INAF), Université Laval, QC G1V 0A6, Canada; 3 Centre de recherche de l’Institut Universitaire de Cardiologie et Pneumologie de Québec, Université Laval, QC G1V 4G5, Canada; 4 Department of Pathology, CHU de Québec - Hôpital Saint-Sacrement, QC G1S 4L8, Canada; 5 Endocrinology and Nephrology Axis, Centre de recherche du CHU de Québec – Université Laval, QC G1V 4G2, Canada

**Keywords:** Omega-3 fatty acids, Eicosapentaenoic acid, Gleason score, High-grade prostate cancer, Active surveillance

## Abstract

Dietary omega-3 fatty acids (ω3), particularly long-chain ω3 (LCω3), have protective effects against prostate cancer (PCa) in experimental studies. Observational studies are conflicting, possibly because of the biomarker used. This study aimed at evaluating associations between grade reclassification and ω3 levels assessed in prostatic tissue, red blood cells (RBC), and diet. We conducted a validation cross-sectional study nested within a phase II clinical trial. We identified 157 men diagnosed with low-risk PCa who underwent a first active surveillance repeat prostate biopsy session. Fatty acid (FA) intake was assessed using a food frequency questionnaire and their levels measured in prostate tissue and RBC. Associations were evaluated using logistic regression. At first repeat biopsy session, 39 (25%) men had high-grade PCa (grade group ≥2). We found that high LCω3-eicosapentaenoic acid (EPA) level in prostate tissue (odds ratio (OR) 0.25; 95% (confidence interval (CI) 0.08–0.79; *p*-trend = 0.03) was associated with lower odds of high-grade PCa. Similar results were observed for LCω3 dietary intake (OR 0.30; 95% CI 0.11-0.83; *p*-trend = 0.02) but no association for RBC. LCω3-EPA levels in the target prostate tissue are inversely associated with high-grade PCa in men with low-risk PCa, supporting that prostate tissue FA, but not RBC FA, is a reliable biomarker of PCa risk.

## 1. Introduction

The worldwide difference in prostate cancer (PCa) incidence highlights an important role of lifestyle in PCa [1,2,3,4]. Dietary fatty acid (FA) intake is one dietary factor thought to impact PCa development. Interestingly, Inuit people have a very low incidence of PCa and pre-malignant lesion at autopsy, which may be attributed to their traditional diet rich in omega-3 FA (ω3) [5,6]. In addition, preclinical and clinical experimental studies support that high ω3 intake have protective effects against PCa, likely via their anti-inflammatory properties [7]. Animal studies showed that ω3, particularly long-chain ω3 (LCω3), suppress neoplastic transformation, angiogenesis, and tumor cell growth [8,9]. A prospective randomized trial with fish oil supplementation showed that LCω3 decrease PCa cell proliferation in men undergoing radical prostatectomy [10,11].

Epidemiological studies provide mixed results regarding associations between FA intake and PCa. Some studies showed that a high level of ω3 is inversely associated with PCa risk [12,13,14,15,16], while other studies demonstrated null [17,18,19,20,21] or positive associations [22,23,24]. These studies used various methods to evaluate FA intake, such as food frequency questionnaires (FFQ) and FA composition of plasma circulating lipids or of red blood cell (RBC) membranes. Most of the studies evaluating the association between ω3 and PCa were based on FFQ [12,13,14,15,19,23,25,26], while fewer studies were based on FA levels in plasma or in RBC membranes [16,18,20,21,22,24]. The FA composition of RBC is thought to be more reliable than plasma, as it reflects the last 3-month diet [27]. To our knowledge, only one previous study was based on FA levels in the prostate tissue [28]. In this cohort of 48 men with low-risk PCa, we measured the level of eicosapentaenoic acid (EPA), a LCω3 subtype, directly in prostatic tissue, and found that it was inversely associated with reclassification to high-grade disease. This association was not observed in RBC, suggesting that information bias may be present when considering only blood FA measures.

We undertook a validation study using the same methodology. We hypothesized that ω3 intake, and particularly LCω3 levels in the target prostate tissue, are inversely associated with high-grade PCa during active surveillance. The ω3 can be assessed through food intake, directly in circulating RBC or in target tissue such as prostate. In this study, we aimed at evaluating the associations between ω3 status assessed through different measurements and high-grade PCa, or grade reclassification, in men under active surveillance for a low-risk PCa.

## 2. Materials and Methods

### 2.1. Study Population

This study is a cross-sectional study of 189 men recruited for a phase II randomized controlled trial. Men were recruited at the time of a low-risk PCa diagnosis for which active surveillance was elected. Low-risk PCa was defined as pathologically confirmed grade group 1, with < 6 positive biopsy cores out of 12, < 3 positive sextants, a clinical stage ≤ T2a, and a prostate-specific antigen (PSA) level < 15 ng/mL if prostate volume was >30 mL, or a PSA < 10ng/mL otherwise. Patients were excluded if they were taking a 5α-reductase inhibitor (5-ARI) drug, ω3 supplements, or if they had received radiotherapy or chemotherapy. The ongoing randomized trial assesses the effects on prostate tissue biomarkers of a dietary intervention versus dutasteride, a 5-ARI drug. The dietary intervention aims at increasing intake of LCω3 while decreasing ω6 FA and *trans*-fat intake.

**Figure 1:**
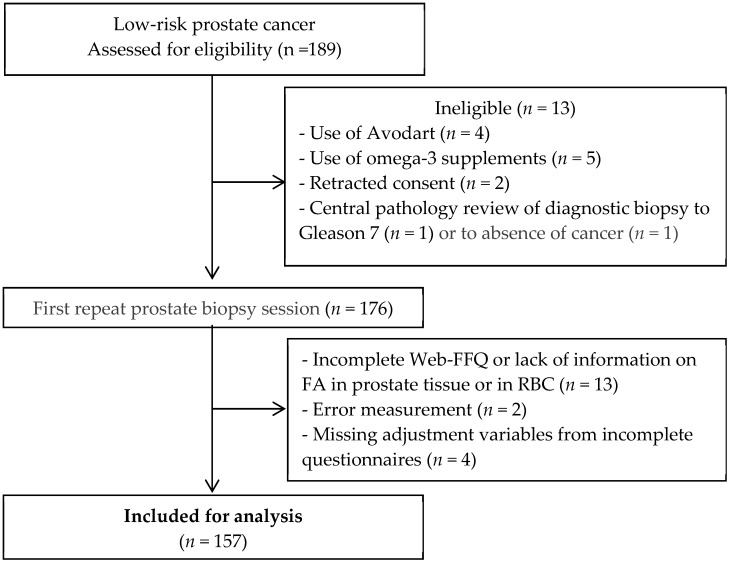
Flow chart of selection for study subjects. 189 men diagnosed with low-grade prostate cancer (PCa) and electing an active surveillance program were recruited for this study. After several months, men underwent a new prostate biopsy session (first repeat, or confirmatory biopsy) to assess PCa grade. Note: Web-FFQ = validated Food Frequency Questionnaire online; FA = Fatty Acids; RBC = Red Blood Cells.

The current cross-sectional study investigates how FA levels are associated with a high-grade PCa (grade group ≥ 2) at the first repeat biopsy, which was performed for randomization into the trial. A planned analysis of the first 48 patients, which amounted to about one-fourth of all patients recruited, has already been published [28]. We present here the data at baseline for the complete study population. From 189 men recruited for this study, 176 were eligible according to exclusion criteria listed in Figure 1. Men were included if information was available for FA intake obtained from FFQ, as well as FA levels in RBC and in prostate tissue (*n* = 157).

All patients provided written informed consent to participate in the study. The protocol is registered to clinicaltrials.gov (NCT01653925). The Centre Hospitalier Universiatire (CHU) de Québec-Université Laval ethics board approved the trial and the current study (2012-466).

### 2.2. Data Collection

#### 2.2.1. PCa Grade

Following the initial diagnosis of low-risk PCa, patients underwent a first repeat (confirmatory) biopsy session performed by the same ultrasonographist (VF), which was planned within 6 months (range 2–14 months) after initial diagnosis. Central pathology review of all biopsy cores was evaluated using the recent WHO-endorsed Gleason grading system, as proposed by Epstein [29,30]. High-grade PCa was defined as Gleason score ≥ 7, which included grade group 2 (Gleason 3+4) or greater.

#### 2.2.2. FA Profiles

FA levels in prostatic tissue and RBC: At first repeat biopsy session, additional prostate biopsy cores were taken in normal peripheral zones of the prostate presenting no prostate nodule, no ultrasound abnormality, and within a sextant region where the initial diagnostic biopsy cores were negative. These biopsy cores were dedicated to research and were immediately placed in cold Hank’s Balanced Salt Solution (HBSS; Invitrogen), frozen in dry ice, and stored at −80 °C. 

Blood samples were collected into dipotassium ethylenediaminetetraacetic acid (K2EDTA)-containing Vacutainer tubes (Becton Dickinson) on the morning of the first repeat biopsy session, after an overnight fast, and immediately placed on ice. Within 1 h, plasma, buffy coat, and RBC were separated and isolated by centrifugation (2500 g, 15 min, 4 °C). Isolated fractions were stored at −80 °C.

FA profiles of normal prostate biopsies and RBC were determined by gas chromatography after extraction of total lipids, as previously described [28,31,32]. The reliability of repeat measurements of FA profiles in prostatic tissue by gas chromatography were previously demonstrated [28]. FA profiles in RBC is expressed as percentage of total FA, while in prostatic tissue as percentage of total FA as well as in milligrams of FA per gram of tissue.

FA intake: Patients were asked to complete a validated quantitative, web-based, self-administered FFQ online (web-FFQ) within a few days of the first repeat biopsy session to assess food intake over the last month. This web-FFQ contains standardized photographs of most food items, to help the participant quantify portion sizes, and was developed and validated specifically for Quebec citizens, who constitute the totality of our study population [33]. We also previously validated ability of this web-FFQ to assess ω3 intake specifically in PCa patients [34].

### 2.3. Confounding Variables

Anthropometric measures were taken according to standardized procedures by research personnel trained specifically for this assessment. Information pertaining to cancer risk factors, such as age, education level, smoking status, medical history, and physical activity, was also collected. Physical activity was measured using the validated Godin-Shephard Leisure-Time Physical Activity Questionnaire [35]. In this questionnaire, duration and intensity components are scored, which allows classification of individuals into three validated categories: active (PA score ≥ 24), moderately active (14 ≤ PA score < 24) and inactive (PA score < 14) [36].

### 2.4. Statistical Analysis

Descriptive statistics for low- and high-grade PCa groups at first repeat biopsy were compared using Wilcoxon test for continuous variables and using chi-squared test for categorical variables.

To estimate associations between FA status (assessed either in prostate tissue, RBC, or diet) and reclassification to high-grade PCa at first repeat biopsy, we categorized FA in tertiles. We fitted logistic regression models to estimate odds ratios (OR) and 95% confidence intervals (95% CI) of high-grade disease across FA tertiles, relative to the lowest tertile. Models were adjusted for potential confounding factors identified from the literature and that are potentially associated with PCa grade (*p* ≤ 0.30 in the multivariable analysis): waist circumference (continuous), PSA (continuous), alcohol (continuous), and physical activity (active, moderately active, and inactive) [36,37]. In addition, age (continuous) and total energy intake (continuous) were included in all models independently of p-values. We performed sensitivity analyzes by adjusting for body mass index (BMI) instead of waist circumference, for prostate volume, and also for education. These sensitivity analyses did not materially change the results. We also conducted a trend test (*p*-trend) by treating the median of tertiles as a continuous variable in multivariable regression models. In order to explore the possibility of residual confounding by using tertiles, we categorized more finely the prostate tissue EPA levels according to their observed distribution (Supplementary Figure S1), showing a group at the inferior limit of sensitivity, a middle group split in half, and high outliers (0; 0–0.2; 0.2–0.4; >0.4). We tested the dose-response effect by using the Mantel-Haenszel test, as is was not possible to normalize the distribution of prostate tissue EPA, precluding linear modeling. Finally, we calculated correlations between FA from web-FFQ, RBC membranes, and prostate tissue using partial Spearman correlations.

All statistical analyses were performed using SAS version 9.4. All tests were two-sided and *p* < 0.05 was considered as statistically significant. In order to evaluate the possibility of selection bias, we compared the characteristics of included and excluded participants.

## 3. Results

We identified 157 men meeting inclusion criteria. At first repeat—or confirmation—biopsy, 39 men were reclassified to high-grade PCa and 118 had low-grade disease. Characteristics of the study population are summarized in Table 1. The mean age of patients was 61 years (± 7) and the mean PSA was 5.0 ng/mL (± 2.7). Of those men, 43% had a university degree and 38% had never smoked. BMI higher than 30 Kg/m^2^ was observed in 23% of participants. The characteristics of the included and excluded patients were similar, except for age (Supplementary Table S1).

Table 2 presents FA dietary intake, FA profiles in RBC, and in prostatic tissue at the first repeat biopsy session. The mean caloric intake was 2500 kcal (± 759). Participants had an average daily intake of ω3 and LCω3 of 2.17g (± 0.90) and 0.41g (± 0.35), respectively. The mean ω3/ω6 ratio from dietary intake was 0.15 (± 0.05) and was borderline higher in low-grade PCa group (*p* = 0.05). This difference was significantly reflected in prostate tissue (*p* < 0.01). Finally, LCω3 subtype EPA level in prostatic tissue was higher in low-grade PCa group (*p* = 0.01).

Associations between FA levels in prostate tissue and reclassification to high-grade PCa are presented in Table 3. FA levels in prostate tissue are associated with high-grade PCa. Compared to men in the lowest tertile of EPA level in prostatic tissue, men in the highest tertile had a decreased risk of high-grade PCa (OR 0.25; 95% CI 0.08-0.79; *p*-trend = 0.03). Weaker and stronger associations were observed, respectively, for the LCω3/ω6 ratio and ω3/ω6 ratio measured in prostatic tissue (OR 0.38; 95% CI 0.14–1.00; *p*-trend = 0.05, and OR 0.20; 95% CI 0.07–0.59; *p*-trend < 0.01, respectively). Table 4 shows the analysis with finer categories of EPA measured in prostate tissue (Supplementary Figure S1), in which we found a significant dose-response association between EPA and high-grade (*p*-trend < 0.01).

The enzymatic activity for long-chain (LC) FA metabolism in prostate tissue was estimated for ω3 and ω6. As shown in Table 5, we found that compared to high-grade, low-grade PCa group had a higher activity of ∆5-desaturase, which converts eicosatetraenoic acid (ETA) to EPA, as well as a lower activity of Elovl2 elongase, which converts EPA to docosapentaenoic acid (DPA), altogether suggesting an EPA bioaccumulation in prostate tissue of low-grade PCa (Supplementary Figure S2).

Associations between FA levels in RBC and reclassification to high-grade PCa are presented in Table 6. We observed a similar direction of effect of the LCω3/ω6 ratio measured in RBC than that measured in prostate tissue, but none of the RBC associations were significant (all *p*-trends > 0.20).

The associations between dietary FA intake and reclassification to high-grade PCa are presented in Table 7. Contrasting with FA levels in RBC, men in the highest tertile of LCω3 intake had a decreased risk of high-grade PCa (adjusted OR 0.30; 95% CI 0.11–0.83; *p*-trend = 0.02) compared to men in the lowest tertile of LCω3 intake. A similar protective effect was observed with a higher LCω3/ω6 ratio (OR 0.29; 95% CI 0.11–0.76; *p*-trend = 0.03).

## 4. Discussion

This is a validation study of the first and only study that evaluated the associations between high-grade PCa and FA content measured in the prostate tissue during active surveillance, moreover linking it to RBC and diet. The opportunity of an ongoing randomized controlled trial in men starting active surveillance was ideally suited to examine that question. We found that high level of EPA, a subtype of LCω3, was associated with a decreased risk of high-grade disease in the prostate tissue, and found a significant dose-response association (*p*-trend < 0.01). We also observed that higher ω3/ω6 and LCω3/ω6 ratios decreased risk of reclassification to high-grade disease. This validation study supports our a priori hypothesis, based on our initial study [28], that higher level of ω3, particularly EPA, is inversely associated with risk of high-grade PCa. This current study represents an important opportunity to re-examine this question under the lens of information bias, given that two recent studies reported conflicting results about the role of ω3 on PCa [18,24]. These observational studies were conducted within large-scale randomized trials testing other interventions on PCa incidence. Such studies are subject to prognostic selection bias, potentially limiting the generalizability of study findings.

More importantly, the prostate gland is metabolically active. Indeed, the estimated prostatic FA metabolism was also linked to high-grade PCa. Compared to the high-grade group, the low-grade group had a higher activity of ∆5-desaturase, which converts ETA to EPA, while the activity of Elovl2 elongase, which converts EPA to DPA, was significantly lower. Thus, the prostatic FA metabolism, which translates to elevated EPA levels, decreased the risk of high-grade disease. This supports our hypothesis that EPA may be a key protector against PCa aggressiveness.

In order to evaluate if the FA measurement in RBC can be used as biomarker for the prostate FA content, and hence a PCa biomarker, we also examined its relation with PCa grade. We observed that none of the FA measured in RBC were significantly associated with PCa grade. Several methodologic distinctions of our study are worth mentioning to support our findings. Our bio-banking protocol included drawing blood in the early morning after overnight fasting, as well as treatment of bio-specimens within one hour of collection to prevent FA oxidation, which affects the accuracy of FA profiles. These procedures were not part of any of the previous blood FA biomarker studies. Moreover, FA profiles measured in RBC are much less affected by the recent dietary intake and circadian variation than is the FA profile measure in plasma phospholipids, which was used in the vast majority of LCω3 biomarkers studies of PCa risk, including the two large-scale recent ones [18,24]. The RBC FA profile rather reflects the FA content of diet of the last three months. One explanation for the lack of association between blood biomarker and PCa grade might also be that the RBCs, being anucleated, reflect both dietary intake and FA metabolism of the whole body, not that specifically of the prostate. In fact, we observed a regression to the mean effect of the same direction of associations with loss of significance. In addition, the FA level correlations between RBC and prostate tissue were weak, at best moderate (Supplementary Table S2). Unfortunately, RBC FA profile does not seem to be a good biomarker for PCa compared to FA profile in the prostatic tissue.

Since LCω3 FA are essential, we also verified the association with ω3 measured in the diet. We observed, in line with the prostate content, that a high dietary content in LCω3 and LCω3/ω6 were associated with a deceased risk of high-grade disease. This contrasts with the absence of association with FA levels in RBC. This suggests that there may be other protector elements in the diet in addition to LCω3 alone. Interestingly, while we found no such data in cancer patients, we found a trial in insulin-resistant patients that have compared the source of ω3. Patients consumed equal amounts of LCω3 directly from fish versus from processed fish oil. Only fish-consumed LCω3 reduced C-reactive protein, a marker of systemic inflammation, and improved insulin sensitivity [38,39]. Thus, the dietary source of LCω3 may matter more for clinical inflammatory-related effects than their blood levels alone. In our current study, even though small sample size precluded most post-hoc subgroup analyses, we observed that greater fish intake (at least once a week) decreased the risk of high-grade PCa (adjusted OR 0.40; 95% CI 0.16–0.98; *p* = 0.04), while intake of plant sources of LCω3, such as flaxseed and other nuts, did not (OR 0.73; 95% CI 0.31–1.72; *p* = 0.47). However, we did not find any association with vitamin D and other protein sources (data not shown).

Our findings of a protective association between LCω3 and high-grade PCa are in line with previous experimental studies. In fact, most preclinical experimental studies in mice showed that a LCω3 enriched diet reduces PCa progression [40,41,42,43,44]. Also, all clinical trials testing the impact of LCω3 on PCa cell proliferation showed a significant reduction [10,11,45]. However, results from observational studies have been inconsistent. Among 30 cohorts and case-control studies investigating incidence of PCa and FA intake or biomarkers thereof, 11 reported inverse associations, similar to our study; the others showed positive or no association. These inconsistent observations may arise from various important methodological issues. First, various techniques and sample types are used to measure FA. For example, the contradictory studies have measured FA concentration in plasma or serum. Plasma levels of ω3 FA are considered a poor biomarker of long-term ω3 intake, because this measurement may be highly influenced by the last meal’s content and plasma triglyceride levels, especially if patients were not fasting prior to blood collection, which was the case in the two larger recent studies [18,24]. These methods can lead to information bias of the exposure measure. Second, also in contrast to our study, most studies did not adjust for many potential confounding factors, such as physical activity, alcohol, and anthropometric measures. All of these may affect systemic and possibly prostate tissue inflammation, a potential causal mechanism of ω3, and other biological pathways leading to PCa progression [37]. Moreover, none of these studies measured the FA profile of the target prostate tissue, supporting the relevance of our study design.

To our knowledge, this is the first study to examine the relation between the FA profile of prostate tissue and high-grade PCa during active surveillance. We found only one other group who examined how prostate tissue FA profile was related to a risk measure of PCa aggressiveness [46]. In radical prostatectomy specimens, prostate tissue levels of LCω3 (EPA + DHA) were inversely associated with the risk of locally advanced disease (OR 0.52, 95% CI, 0.30-0.90; *p* = 0.02) [46]. There is currently no non-invasive method to quantify any tissue FA profile, and we are the first group to examine this profile while the prostate is still at risk of cancer progression. With emerging technologies, the concept of measuring metabolic aspects of prostate tissue non-invasively is becoming a reality. For example, magnetic resonance spectroscopy provides a non-invasive method of detecting small molecular biomarkers (choline-containing metabolites, polyamines, and citrate) within the prostate [47,48]. This improves diagnosis and staging of PCa [47,48], and algorithms are being derived to identify prostate tissue inflammation [49,50,51].

This prostate tissue chronic inflammation is an increasingly supported causal mechanism of PCa development and progression [52,53]. While ω6 are pro-inflammatory, LCω3 are anti-inflammatory, mainly because of their derived metabolites [54]. These include eicosanoids, which also have anti-neoplastic properties when deriving from LCω3 [55]. A case-control study in the placebo arm of the Prostate Cancer Prevention Trial (PCPT) observed that, as expected from studies of systemic markers of inflammation, plasma ω3 were inversely associated to prostate tissue inflammation [56]. This observation supports the findings of our current study. However, another study by Brasky et al. observed in the same PCPT study [18] found positive associations between plasma ω3 FA levels and risk of PCa diagnosis. Thus, because of conflicting observational evidence, PCa risk cannot clearly be related to blood markers of LCω3 intake.

Some other methodological aspects of our study are worth mentioning. We used a cross sectional design, nested in a randomized trial, in which many methodological aspects were designed to evaluate the role of dietary FA on PCa grade. First, dietary intake was measured before the outcome ascertained, reducing possibility of recall bias, which affects some case-control studies of dietary factors. Second, the widely used and well-validated web-FFQ used in the present study measures various food preparation aspects, not measured in many FFQ, but important in FA biology, such as type of oil dressing and cooking modes. This is a great opportunity to evaluate the impact of FA on PCa. Third, rather than a risk of diagnosis model where causal factors may act during a very long period, we used an active surveillance model. This model provides an opportunity to examine the last factors leading to high-grade, potentially lethal, PCa. Inflammation could play an important role in that transition. However, some data provide uncertainty to this chain of continuity [57,58], suggesting that low-grade and higher-grade tumor nodules are different entities. Finally, in this current study, the same specialized ultrasonographist performed all study imaging procedures and tissue sampling, using a specific protocol uniform throughout the study. This eliminates inter-observer variation and contributes to increase internal validity.

In summary, this study validates in a larger cohort our previous findings suggesting that LCω3, the EPA subtype in particular, are protective against high-grade PCa. Specifically, the prostate tissue level of EPA and the LCω3/ω6 ratio were inversely associated with high-grade PCa at initiation of active surveillance in men with low-risk PCa. The dietary intake of LCω3, essential FA, and the LCω3/ω6 ratio showed similarly protective associations. In contrast, we observed both an absence of associations between grade and the RBC FA profile, and weak correlations between RBC and prostate tissue FA profiles, suggesting that blood FA biomarkers may be less useful in their capacity to predict PCa aggressiveness. This study provides a rationale for future PCa prevention studies to increase LCω3 intake from food rather than from processed supplement sources. More studies are needed and justified to decipher the effects of LCω3 on PCa, as well as to identify the best FA biomarker of PCa risk and aggressiveness.

## Figures and Tables

**Table 1:** Characteristics of the study subjects at the first repeat biopsy session.

Variables	Total (*n* = 157)	Low-Grade (*n* = 118)	High-Grade (*n* = 39)	*p*-Value ^*^
**Age (y)**				0.84
Mean ± SD	61.3 ± 7.4	61.3 ± 7.2	61.2 ± 8.2	
Median (Q1–Q3)	62.0 (56.0–67.0)	62.0 (56.0–67.0)	62.0 (56.0–67.0)	
**PSA (ng/mL)**				0.26
Mean ± SD	5.0 ± 2.7	5.04 ± 2.9	5.05 ± 2.05	
Median (Q1–Q3)	4.7 (3.3–6.0)	4.6 (3.0–6.2)	4.8 (4.0–5.9)	
**Waist Circumference (cm)**				0.42
Mean ± SD	90.0 ± 10.2	95.2 ± 9.3	98.5 ± 12.5	
Median (Q1–Q3)	96.0 (89.0–102.0)	95.0 (88.0–101.0)	96.0 (89.0–105.0)	
**BMI Kg/m^2^, n (%)**				0.26
<25	44 (28.39)	35 (29.66)	9 (24.32)	
25–30	76 (49.03)	60 (50.85)	16 (43.24)	
>30	35 (22.58)	23 (19.49)	12 (32.43)	
**Education level attained, *n* (%)**				0.34
Secondary school or less	46 (29.30)	31 (26.27)	15 (38.46)	
Postsecondary diploma	44 (28.03)	34 (28.81)	10 (25.64)	
University degree	67 (42.68)	53 (44.92)	14 (35.90)	
**Smoking status, *n* (%)**				0.91
Current smoker	10 (6.37)	7 (5.93)	3 (7.69)	
Former smoker	88 (56.05)	66 (55.93)	22 (56.41)	
Never	59 (37.58)	45 (38.14)	14 (35.90)	
**Physical activity score, *n* (%)**				0.33
Active	94 (59.87)	74 (62.71)	20 (51.28)	
Moderately active	13 (8.28)	8 (6.78)	5 (12.82)	
Inactive	50 (31.85)	36 (30.51)	14 (35.90)	

**NOTE**: In this study, 157 men diagnosed with low–risk prostate cancer (PCa) and managed under active surveillance underwent a first repeat biopsy session. At the first repeat biopsy session, 39 men out of 157 had a high-grade PCa (grade group ≥ 2). Note: * = *p*-values were obtained using the Wilcoxon test for continuous variables and chi-2 test for categorical variables. SD= standard deviation; Q1 = lower quartile; Q3 = upper quartile; PSA = prostate-specific antigen; BMI = body mass index.

**Table 2:** Fatty acid intake, fatty acid profiles of RBC membranes, and of prostate tissue stratified by prostate cancer grade.

Method	Variables	Total *n* = 157	Low-Grade *n* = 118	High-Grade *n* = 39	*p*-Value ^§^
Web-FFQ *	**Energy (kcal)**				0.23
	Mean ± SD	2496 ± 759	2468 ± 755	2583 ± 775	
	Median (Q1–Q3)	2449 (1901–2952)	2416 (1871–2869)	2604 (1991–2975)	
	**Alcohol (g)**				0.42
	Mean ± SD	16.80 ± 16.92	15.77 ± 15.67	19.93 ± 20.16	
	Median (Q1–Q3)	12.73 (4.70–21.15)	11.32 (4.70–21.49)	14.94 (4.68–20.30)	
	**Total fat (g)**				0.08
	Mean ± SD	94.17 ± 35.33	91.89 ± 35.69	101.05 ± 33.77	
	Median (Q1–Q3)	89.87 (69.68–113.81)	87.51 (68.86–107.90)	97.99 (74.64–121.04)	
	**Total ω3**				0.38
	Mean ± SD	2.17 ± 0.90	2.16 ± 0.96	2.20 ± 0.70	
	Median (Q1–Q3)	2.00 (1.64–2.53)	1.96 (1.61–2.52)	2.07 (1.64–2.79)	
	**ALA**				0.09
	Mean ± SD	1.76 ± 0.73	1.72 ± 0.75	1.87 ± 0.67	
	Median (Q1–Q3)	1.64 (1.30–2.12)	1.58 (1.28–2.02)	1.84 (1.35–2.23)	
	**LCω3**				0.09
	Mean ± SD	0.41 ± 0.35	0.44 ± 0.38	0.32 ± 0.26	
	Median (Q1–Q3)	0.32 (0.17–0.57)	0.35 (0.19–0.62)	0.22 (0.16–0.44)	
	**EPA**				0.15
	Mean ± SD	0.15 ± 0.15	0.16 ± 0.16	0.11 ± 0.11	
	Median (Q1–Q3)	0.11 (0.05–0.19)	0.12 (0.05–0.20)	0.08 (0.05–0.16)	
	**DHA**				0.08
	Mean ± SD	0.22 ± 0.19	0.24 ± 0.20	0.18 ± 0.14	
	Median (Q1–Q3)	0.17 (0.09–0.29)	0.19 (0.11–0.33)	0.12 (0.09–0.25)	
	**DPA**				0.17
	Mean ± SD	0.04 ± 0.04	0.04 ± 0.04	0.03 ± 0.03	
	Median (Q1–Q3)	0.03 (0.02–0.06)	0.03 (0.02–0.06)	0.03 (0.02–0.04)	
	**Total ω6**				**0.03**
	Mean ± SD	14.91 ± 6.37	14.23 ± 6.10	16.96 ± 6.80	
	Median (Q1–Q3)	13.47 (10.36–18.64)	13.25 (10.18–17.05)	17.28 (10.62–22.00)	
	**ω3/ω6 ratio**				0.05
	Mean ± SD	0.15 ± 0.05	0.16 ± 0.05	0.14 ± 0.04	
	Median (Q1–Q3)	0.15 (0.12–0.18)	0.16 (0.12–0.18)	0.14 (0.11–0.16)	
	**LCω3/ω6 ratio**				**0.02**
	Mean ± SD	0.03 ± 0.03	0.03 ± 0.03	0.02 ± 0.01	
	Median (Q1–Q3)	0.02 (0.01–0.04)	0.03 (0.01–0.04)	0.02 (0.01–0.03)	
**RBC ^†^**	**Total ω3**				0.43
	Mean ± SD	7.98 ± 1.42	8.04 ± 1.47	7.80 ± 1.26	
	Median (Q1–Q3)	7.75 (6.99–8.80)	7.76 (7.06–8.80)	7.75 (6.91–8.82)	
	**ALA**				0.30
	Mean ± SD	0.12 ± 0.08	0.12 ± 0.08	0.10 ± 0.07	
	Median (Q1–Q3)	0.14 (0.00–0.17)	0.14 (0.00–0.17)	0.14 (0.00–0.16)	
	**LCω3**				0.52
	Mean ± SD	7.79 ± 1.42	7.86 ± 1.50	7.60 ± 1.13	
	Median (Q1–Q3)	7.57 (6.80–8.58)	7.57 (6.93–8.63)	7.35 (6.71–8.54)	
	**EPA**				0.19
	Mean ± SD	0.86 ± 0.43	0.88 ± 0.46	0.77 ± 0.27	
	Median (Q1–Q3)	0.79 (0.60–0.98)	0.81 (0.60–0.99)	0.72 (0.61–0.89)	
	**DHA**				0.70
	Mean ± SD	4.22 ± 0.93	4.24 ± 0.98	4.13 ± 0.79	
	Median (Q1–Q3)	4.07 (3.59–4.75)	4.10 (3.59–4.89)	4.05 (3.50–4.67)	
	**DPA**				0.76
	Mean ± SD	2.72 ± 0.38	2.73 ± 0.39	2.69 ± 0.33	
	Median (Q1–Q3)	2.65 (2.46–2.91)	2.66 (2.43–2.91)	2.64 (2.52–2.85)	
	**Total ω6**				0.21
	Mean ± SD	28.52 ± 1.67	28.40 ± 1.79	28.90 ± 1.18	
	Median (Q1–Q3)	28.76 (27.75–29.65)	28.66 (27.48–29.55)	28.82 (28.16–29.86)	
	**ω3/ω6 ratio**				0.30
	Mean ± SD	0.28 ± 0.07	0.29 ± 0.07	0.27 ± 0.05	
	Median (Q1–Q3)	0.27 (0.24–0.31)	0.27 (0.24–0.31)	0.26 (0.23–0.31)	
	**LCω3/ω6 ratio**				0.36
	Mean ± SD	0.27 ± 0.07	0.28 ± 0.07	0.26 ± 0.05	
	Median (Q1–Q3)	0.26 (0.23–0.31)	0.26 (0.24–0.31)	0.25 (0.22–0.31)	
**Prostate tissue ^‡^**	**Total ω3**				0.05
	Mean ± SD	3.52 ± 1.01	3.62 ± 1.05	3.23 ± 0.81	
	Median (Q1–Q3)	3.36 (2.75–4.13)	3.52 (2.76–4.20)	3.08 (2.57–3.84)	
	**ALA**				0.40
	Mean ± SD	0.45 ± 0.33	0.44 ± 0.33	0.48 ± 0.33	
	Median (Q1–Q3)	0.46 (0.17–0.69)	0.43 (0.17–0.65)	0.57 (0.16–0.73)	
	**LCω3**				0.13
	Mean ± SD	2.75 ± 1.13	2.84 ± 1.17	2.50 ± 0.98	
	Median (Q1–Q3)	2.70 (1.90–3.40)	2.76 (1.96–3.50)	2.29 (1.82–3.22)	
	**EPA**				**0.01**
	Mean ± SD	0.14 ± 0.15	0.16 ± 0.16	0.09 ± 0.08	
	Median (Q1–Q3)	0.13 (0.00–0.20)	0.14 (0.05–0.21)	0.10 (0.00–0.15)	
	**DHA**				0.20
	Mean ± SD	1.76 ± 0.80	1.81 ± 0.81	1.61 ± 0.75	
	Median (Q1–Q3)	1.75 (1.10–2.35)	1.83 (1.10–2.39)	1.44(1.03–2.21)	
	**DPA**				0.20
	Mean ± SD	0.85 ± 0.31	0.87 ± 0.32	0.80 ± 0.28	
	Median (Q1–Q3)	0.82 (0.60–0.98)	0.83 (0.61–0.98)	0.72 (0.60–1.00)	
	**Total ω6**				0.95
	Mean ± SD	21.97 ± 3.74	21.98 ± 3.70	21.97 ± 3.89	
	Median (Q1–Q3)	22.10 (19.19–24.25)	22.32 (19.19–24.15)	21.69 (18.89–25.96)	
	**ω3/ω6 ratio**				**<0.01**
	Mean ± SD	0.16 ± 0.04	0.17 ± 0.04	0.15 ± 0.02	
	Median (Q1–Q3)	0.16 (0.14–0.17)	0.16 (0.14–0.18)	0.14 (0.13–0.16)	
	**LCω3/ω6 ratio**				**0.04**
	Mean ± SD	0.12 ± 0.04	0.13 ± 0.04	0.11 ± 0.03	
	Median (Q1–Q3)	0.12 (0.10–0.14)	0.12 (0.10–0.14)	0.11 (0.09–0.13)	

Abbreviations: LCω3 = long-chain omega-3 fatty acids (EPA + DPA + DHA); EPA = Eicosapentaenoic acid; DPA = Docosapentaenoic acid; DHA = Docosahexaenoic acid; ALA = alpha-Linolenic acid. Note: * = Fatty acids are expressed as a daily intake (g); † = fatty acids are expressed as % of total fatty acids in RBC membranes; ‡ = fatty acids are expressed as % of total fatty acids in prostate tissue; § = *p*-values were obtained using the Wilcoxon test. ω3 = omega-3; ω6 = omega-6. Bold font indicates significance at *p* < 0.05.

**Table 3:** Associations between fatty acid profiles of prostate tissue and high-grade prostate cancer.

		Fatty Acids (%) *			Fatty Acids (mg/g) ^†^		
Fatty Acid	Tertile	*n* High-Grade/ *n* Low-Grade	Multivariable Models ^‡^ OR (95% CI) *p*-Value ^§^		*n* High-Grade/ *n* Low-Grade	Multivariable Models ^‡^ OR (95% CI) *p*-Value ^§^	
**ω3 total**	1	15/38	1		16/39	1	
	2	16/37	1.24 (0.50–3.07)	0.64	10/40	0.68 (0.26–1.81)	0.44
	3	8/43	0.49 (0.18–1.36)	0.17	13/39	0.79 (0.31–2.02)	0.63
			p–trend ^||^	0.15			0.68
**ALA**	1	14/37	1		13/40	1	
	2	8/46	0.39 (0.14–1.12)	0.08	11/41	0.92 (0.35–2.41)	0.86
	3	17/35	1.35 (0.54–3.42)	0.51	15/37	1.21 (0.48–3.07)	0.68
			*p*-trend ^||^	0.68			0.65
**LCω3**	1	17/37	1		14/37	1	
	2	11/40	0.68 (0.26–1.78)	0.44	16/39	1.03 (0.41–2.57)	0.95
	3	11/41	0.59 (0.23–1.51)	0.27	9/42	0.59 (0.21–1.65)	0.31
			*p*-trend ^||^	0.27			0.28
**EPA**	1	16/37	1		15/31	1	
	2	18/36	1.35 (0.55–3.31)	0.50	14/28	1.02 (0.39–2.69)	0.95
	3	5/45	**0.25 (0.08–0.79)**	**0.02**	10/59	**0.35 (0.13–0.92)**	**0.03**
			*p*-trend ^||^	**0.03**			**0.03**
**DHA**	1	16/37	1		13/39	1	
	2	11/41	0.78 (0.29–2.08)	0.62	16/38	1.43 (0.57–3.62)	0.45
	3	12/40	0.74 (0.29–1.90)	0.54	10/41	0.74 (0.27–2.10)	0.58
			*p*-trend ^||^	0.54			0.55
**DPA**	1	16/36	1		17/34	1	
	2	12/40	0.73 (0.29–1.86)	0.24	11/40	0.57 (0.22–1.48)	0.25
	3	11/42	0.59 (0.23–1.53)	0.28	11/44	0.50 (0.19–1.27)	0.14
			*p*-trend ^||^	0.28			0.17
**ω6 total**	1	13/40	1		16/37	1	
	2	12/38	1.35 (0.49–3.74)	0.55	6/46	0.32 (0.11–0.96)	0.04
	3	14/40	1.28 (0.48–3.40)	0.62	17/35	1.14 (0.47–2.77)	0.77
			*p*-trend ^||^	0.64			0.54
**Ratio**	1	19/34	1		19/34	1	
**ω3/ω6**	2	14/39	0.55 (0.22–1.34)	0.19	13/40	0.50 (0.20–1.24)	0.13
	3	6/45	**0.20 (0.07–0.59)**	**<0.01**	7/44	**0.24 (0.09–0.68)**	**<0.01**
			*p*-trend ^||^	**<0.01**			**<0.01**
**Ratio**	1	17/36	1		17/36	1	
**LCω3/ω6**	2	13/39	0.73 (0.29–1.86)	0.51	13/39	0.79 (0.31–2.00)	0.62
	3	9/43	0.38 (0.14–1.00)	0.05	9/43	0.42 (0.16–1.10)	0.07
			*p*-trend ^||^	0.05			0.07

**NOTE**: Logistic regression models in which outcome is the presence of high-risk prostate cancer defined as grade group ≥ 2 at the first repeat biopsy session versus low-risk prostate cancer. Note: * = Prostatic fatty acids were expressed as % of total fatty acids of prostatic biopsy; † = prostatic fatty acids were expressed in absolute concentrations (mg of fatty acid per g of prostatic biopsy); ‡ = multivariable models were adjusted for age, prostate-specific antigen (PSA) level, waist circumference, physical activity, alcohol, and total energy intake; § = p-value of category relative to referent (lowest tertile); || = adjusted trend *p*-value. Abbreviations: EPA = Eicosapentaenoic acid; DPA = Docosapentaenoic acid; DHA = Docosahexaenoic acid; ALA = alpha-Linolenic acid; Long-chain ω3 (LCω3) = EPA + DHA + DPA. OR = odds ratio; CI = confidence interval. Bold font indicates significance at *p* < 0.05.

**Table 4:** Trend analysis of EPA level in prostate tissue.

EPA Level *	*n*	Frequency, n (%)		*p*-Trend ^†^	Adjusted *p*-Trend ^‡^
		Low-Grade	High-Grade		
0	44	29 (66%)	15 (34%)	**<0.01**	**0.01**
0–0.2	73	52 (71%)	21 (29%)		
0.2–0.4	33	30 (91%)	3 (9%)		
≥0.4	7	7 (100%)	0 (0%)		

Note: * = EPA level is expressed as % of EPA on total FA of prostate tissue; † = *p*-trend was calculated using Mantel Haenszel test; ‡ = Adjusted *p*-trend was obtained by treating the median of each category as a continuous variable in multivariable regression model adjusted for age, PSA level, waist circumference, physical activity, alcohol, and total energy intake. The two last categories were merged together to bypass the complete separation issue for logistic regression. Bold font indicates significance at *p* < 0.05.

**Table 5:** Estimation of elongase and desaturase activity in high- and low-grade PCa groups.

Fatty Acid Ratio	Fatty Acid Ratio	Enzymatic Activity	mean ± SD		*p*-Value *
			High-Grade	Low-Grade	
20:3n6/18:3n6	DGLA/GLA	Elovl5 elongase	14.07 ± 6.06	15.27 ± 5.14	0.42
22:4n6/20:4n6	Adrenic/AA	Elovl2 elongase	0.16 ± 0.03	0.15 ± 0.04	0.13
20:4n3/18:4n3	ETA/Stearidonic	Elovl5 elongase	0.61 ± 0.35	0.76 ± 0.50	0.77
**22:5n3/20:5n3**	**DPA/EPA**	**Elovl2 elongase**	**6.00 ± 1.68**	**5.32 ± 2.14**	**0.01**
18:1n9/18:0	Oleic/Stearic	∆9-desaturase	3.22 ± 1.96	2.90 ± 1.86	0.42
18:3n6/18:2n6	GLA/LA	∆6-desaturase	0.01 ± 0.01	0.01 ± 0.01	0.50
18:4n3/18:3n3	Stearidonic/ALA	∆6-desaturase	0.82 ± 0.81	0.84 ± 0.88	0.58
20:4n6/20:3n6	AA/DGLA	∆5-desaturase	9.80 ± 21.21	4.86 ± 1.17	0.36
**20:5n3/20:4n3**	**EPA/ETA**	**∆5-desaturase**	**1.32 ± 0.73**	**1.72 ± 1.07**	**0.02**

Abbreviations: DGLA = Dihomo-gamma linolenic acid dihomo-gamma; GLA = gamma linolenic acid; ETA = Eicosatetraenoic acid; DPA = Docosapentaenoic acid; ALA = Alpha-linolenic acid; AA = Arachidonic acid; LA = Linoleic acid. Bold font indicates significance at *p* < 0.05. * = *p*-values were obtained using the Wilcoxon test.

**Table 6:** Associations between fatty acid profiles of RBC and high-grade prostate cancer.

Fatty Acid	Tertile	*n* High-Grade/ *n* Low-Grade	Multivariable Models *		
			OR (95% CI)	*p*-Value ^†^	*p*-Trend ^‡^
**ω3 total**	1	17/36	1		0.24
	2	11/42	0.52 (0.20–1.31)	0.16	
	3	11/40	0.58 (0.23–1.48)	0.25	
**ALA**	1	14/37	1		0.68
	2	17/43	1.23 (0.50–3.00)	0.65	
	3	8/38	0.60 (0.23–1.83)	0.42	
**LCω3**	1	15/37	1		0.43
	2	13/41	0.70 (0.27–1.76)	0.44	
	3	11/40	0.68 (0.26–1.76)	0.42	
**EPA**	1	12/39	1		0.33
	2	18/37	1.46 (0.58–3.65)	0.42	
	3	9/42	0.61 (0.21–1.73)	0.35	
**DHA**	1	13/40	1		0.50
	2	16/37	1.11 (0.44–2.81)	0.82	
	3	10/41	0.73 (0.27–1.99)	0.54	
**DPA**	1	10/43	1		0.93
	2	19/33	2.85 (1.06–7.70)	0.04	
	3	10/42	1.18 (0.42–3.36)	0.75	
**ω6 total**	1	9/43	1		0.20
	2	15/37	1.95 (0.72–5.32)	0.18	
	3	15/38	1.92 (0.70–5.26)	0.20	
**Ratio ω3/ω6**	1	16/37	1		0.23
	2	13/40	0.70 (0.28–1.76)	0.45	
	3	10/41	0.55 (0.21–1.45)	0.22	
**Ratio LCω3/ω–6**	1	15/38	1		0.24
	2	14/39	0.80 (0.32–2.00)	0.64	
	3	10/41	0.56 (0.21–1.49)	0.25	

Note: Logistic regression models in which outcome is the presence of high-risk prostate cancer defined as grade group ≥ 2 at the first repeat biopsy session versus low-risk prostate cancer; * = Multivariable models were adjusted for age, PSA level, waist circumference, physical activity, alcohol, and total energy intake; † = p-value of category relative to referent (lowest tertile); ‡ = adjusted trend p-value; EPA = Eicosapentaenoic acid; DPA = Docosapentaenoic acid; DHA = Docosahexaenoic acid; ALA = alpha-Linolenic acid; Long-chain ω3 (LCω3) = EPA + DHA + DPA.

**Table 7:** Associations between fatty acid intake assessed by the web-FFQ and high-grade prostate cancer.

Fatty Acid	Tertile	*n* High-Grade/ *n* Low-Grade	Multivariable Models *		
			OR (95% CI)	*p*-Value ^†^	*p*-Trend ^‡^
**ω3 total**	1	11/43	1		0.75
	2	15/39	1.69 (0.61–4.70)	0.31	
	3	13/36	1.40 (0.39–5.02)	0.60	
**ALA**	1	12/42	1		0.17
	2	10/43	0.99 (0.32–3.05)	0.99	
	3	17/33	2.29 (0.58–9.07)	0.24	
**LCω3**	1	19/34	1		**0.02**
	2	11/43	**0.38(0.15–0.98)**	**0.04**	
	3	9/41	**0.30 (0.11–0.83)**	**0.02**	
**EPA**	1	16/37	1		0.13
	2	13/41	0.63 (0.25–1.60)	0.33	
	3	10/40	0.46 (0.17–1.26)	0.13	
**DHA**	1	18/35	1		**0.02**
	2	13/41	0.52 (0.20–1.30)	0.16	
	3	8/42	**0.29 (0.10–0.83)**	**0.02**	
**DPA**	1	14/39	1		0.12
	2	17/37	1.05 (0.43–2.58)	0.90	
	3	8/42	0.47 (0.17–1.32)	0.15	
**ω6 total**	1	13/40	1		**0.04**
	2	5/46	0.33 (0.10–1.13)	0.08	
	3	21/32	2.22 (0.64–7.78)	0.20	
**Ratio ω3/ω6**	1	17/36	1		0.17
	2	13/40	0.75 (0.31–1.87)	0.55	
	3	9/42	0.50 (0.19–1.36)	0.18	
**Ratio LCω3/ω6**	1	21/32	1		**0.03**
	2	9/43	**0.27 (0.10–0.71)**	**<0.01**	
	3	9/43	**0.29 (0.11–0.76)**	**0.01**	

Note: Logistic regression models in which outcome is the presence of high-risk prostate cancer defined as grade group ≥ 2 at the first repeat biopsy session versus low-risk prostate cancer; * = Multivariable models were adjusted for age, PSA level, waist circumference, physical activity, alcohol. and total energy intake; † = p-value of category relative to referent (lowest tertile); ‡ = adjusted trend p-value; EPA = Eicosapentaenoic acid; DPA = Docosapentaenoic acid; DHA = Docosahexaenoic acid; ALA = alpha-Linolenic acid; Long-chain ω3 (LCω3) = EPA + DHA + DPA. Bold font indicates significance at *p* < 0.05.

## Supplementary Materials

The following are available online at https://www.mdpi.com/2072-6643/11/7/1616/s1. Table S1: Characteristics of excluded and included participants. Table S2: Correlations between fatty acid intake, fatty acid profiles of RBC membranes, and of prostate tissue. Figure S1: Distribution of eicosapentaenoic acid (EPA) level in prostate tissue. Figure S2: Schematic representation of long-chain polyunsaturated omega-6 and omega-3 fatty acids metabolism.
