# Supplementary material for: Omega-3 Fatty Acids Survey in Men under Active Surveillance for Prostate Cancer: from Intake to Prostate Tissue Level
Source: Nutrients. 2019 Jul 16;11(7):1616. doi: 10.3390/nu11071616 (PMC6683032; doi:10.3390/nu11071616)
Supplement: Supplementary file 1 [file nutrients-11-01616-s001.pdf]

Supplementary Materials (Moussa *et al.*)

Table S1: Characteristics of excluded and included participants

Variable	Included participants (n= 157)	Excluded participants (n= 19)	p-value*
**Age (y)**			0.03
Mean ± SD	61.29 ± 7.41	65.05 ± 5.99	
**Waist circumference (cm)**			0.71
Mean ± SD	96.04 ± 10.25	95.00 ± 11.83	
**PSA (ng/ml)**			0.16
Mean ± SD	5.04 ± 2.71	4.05 ± 1.97	
**Education level, n (%)**			0.43
Secondary school or less	45 (28.85)	5 (33.33)	
Postsecondary diploma	45 (28.85)	2 (13.33)	
University degree	66 (42.31)	8 (53.33)	
**Smoking status, n (%)**			0.63
Current smoker	10 (6.41)	1 (6.25)	
Former smoker	87 (55.77)	7 (43.75)	
Never	59 (37.82)	8 (50.00)	
**BMI, n (%)**			0.70
< 25	44 (28.21)	5 (35.71)	
25 – 30	76 (48.72)	7 (50.00)	
> 30	36 (23.08)	2 (14.29)	
**Physical activity score, n (%)**			0.30
Active	94 (59.87)	5 (38.46)	
Moderately active	13 (8.28)	2 (15.38)	
Inactive	50 (31.85)	6 (46.15)	

* p-values were obtained by using the Wilcoxon test for continuous variables and chi-2 test for categorical variables.

Table S2. Correlations between fatty acid intake, fatty acid profiles of RBC membranes and of prostate tissue.

Fatty acid	Intake vs RBC		Intake vs prostate		RBC vs prostate	
	R	p-value	R	p-value	R	p-value
**ω3 total**	0.44	<0.01	0.24	<0.01	0.27	<0.01
**LCω3**	0.59	<0.01	0.27	<0.01	0.26	<0.01
**ω6 total**	-0.11	0.18	0.03	0.72	0.23	<0.01
**Ratio LCω3/ω6**	0.53	<0.01	0.40	<0.01	0.45	<0.01

NOTE: Spearman's correlations were adjusted for age, waist circumference and energy intake.

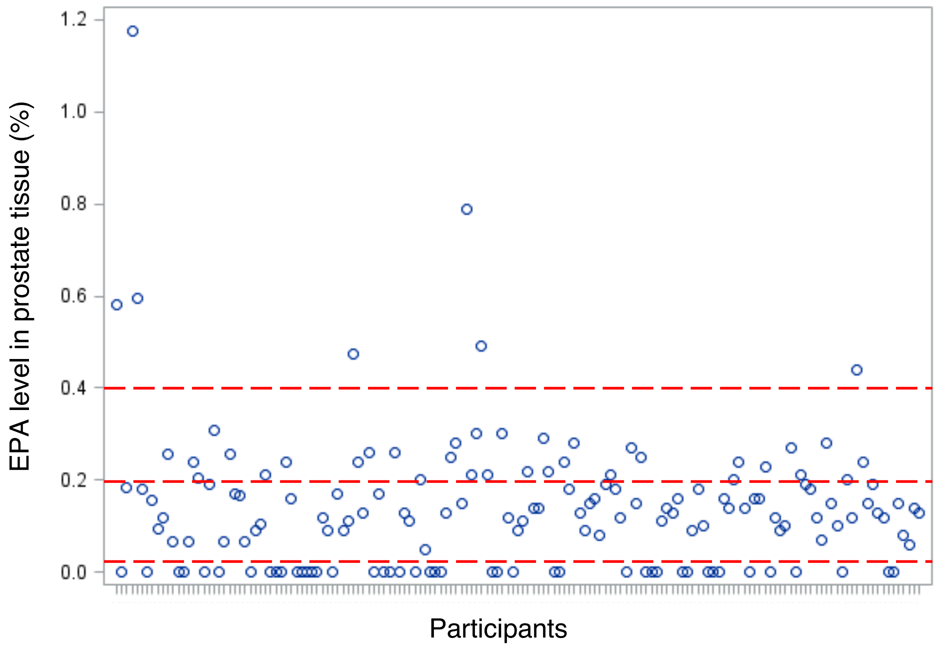

Figure S1. Distribution of eicosapentaenoic acid (EPA) level in prostate tissue. The percentage of EPA level from total fatty acids was used to evaluate a dose-response between EPA level and grade of prostate cancer. EPA was categorized based on observed distribution (dashed red lines). Each dot represents one participant.

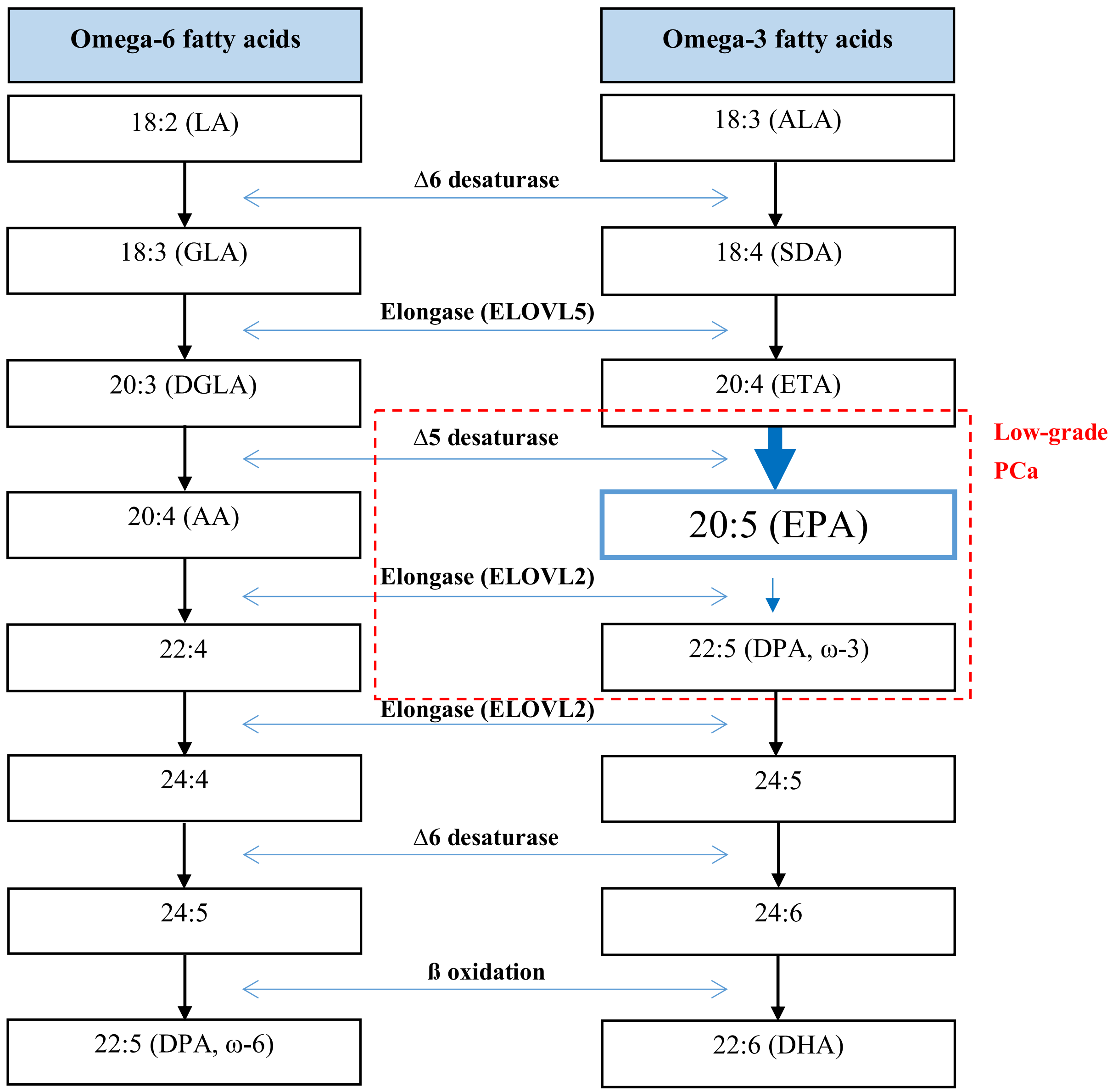

Figure S2. Schematic representation of long-chain polyunsaturated omega-6 and omega-3 fatty acids metabolism. Fatty acids and enzymes are indicated on the graph. High ratio of EPA/ETA and low ratio of DPA/EPA in low-grade PCa suggest a bioaccumulation of EPA in prostate tissue (dashed red line). LA: Linoleic acid, GLA: Gamma linolenic acid, DGLA: Dihomo-gamma linolenic acid dihomo-gamma, AA: Arachidonic acid, DPA: Docosapentaenoic acid, ALA: Alpha-linolenic acid, SDA: Stearidonic acid, ETA: Eicosatetraenoic acid, EPA: Eicosapentaenoic acid, DHA: docosahexaenoic acid.
